# The role of glucose lowering agents on restenosis after percutaneous coronary intervention in patients with diabetes mellitus

**DOI:** 10.1186/1475-2840-8-41

**Published:** 2009-07-28

**Authors:** Chris PH Lexis, Braim M Rahel, Joan G Meeder, Felix Zijlstra, Iwan CC van der Horst

**Affiliations:** 1 Department of Cardiology, VieCuri Medical Centre, Venlo, The Netherlands; 2 Department of Cardiology, University Medical Centre Groningen, Groningen, The Netherlands

## Abstract

**Introduction:**

The prevalence of diabetes is increasing rapidly, and individuals with diabetes are at high risk for cardiovascular disorders. Subsequently the percentage of patients with diabetes subjected to revascularisation, i.e. either percutaneous coronary intervention (PCI) or coronary artery bypass grafting (CABG) also rises rapidly. The outcome of patients with diabetes after PCI is worse than for patients without diabetes. Restenosis is the main limiting factor of the long-term success of PCI. Although stents and antithrombotics improved outcome after PCI in both diabetics and non-diabetics, diabetics still have a worse prognosis. This leads to the suggestion that the restenosis mechanism in diabetics might be different from that in non-diabetics.

**Conclusion:**

Several glucose lowering agents have been shown to influence the restenosis process and thus the outcome after PCI. Current data of especially metformin and thiazolidinediones indicate beneficial results as compared to insulin and sulfonylurea on restenosis. However, no large trials have been undertaken in which the effect of glucose lowering agents on restenosis is associated with improved outcome.

The purpose of this review is to summarize the effect of diabetes and glucose lowering agents on restenosis.

## Introduction

In patients with diabetes, coronary artery disease (CAD) is often present [1]. In comparison with patients without diabetes, CAD is manifest earlier in life [1]. Especially women with diabetes have a significantly higher risk of developing CAD in comparison with men [2]. The calibre of "normal" coronary arteries at coronary angiography is smaller in patients with diabetes [3]. Diabetics have more multi vessel coronary artery disease and form less often collaterals [4,5]. Not only have patients with diabetes more plaques, the plaques are more inflamed and necrotic, forming a risk factor for rupture and therefore myocardial infarction (MI) [6]. After MI diabetics have a worse prognosis [7,8]. Patients with type 2 diabetes without known CAD have a risk of a MI comparable to non-diabetics with a previous history of MI [1]. It is therefore reasonable that CAD is accountable for 70–80% of the diabetes related mortality [7]. Despite improvement of coronary interventions, outcome in patients with diabetes is impaired [1,8]. Restenosis is the main limiting factor of the long-term success of percutaneous coronary interventions (PCI) [9]. Possibly the restenosis mechanism in diabetics might be different from that in non-diabetics. The purpose of this review is to summarize the influence of diabetes on coronary restenosis and to summarize the effect of glucose lowering agents on restenosis and outcome.

## Mechanism of Disease

### Restenosis in diabetics

Diabetes is one of the most important risk factors for restenosis after stent implantation, with odds ratios of 1.9 to 2.5 [10]. Diabetes leads to more extensive neointimal hyperplasia, plaque formation, altered hemodynamics and inadequate compensatory remodeling (table 1) [8]. Restenotic intimal hyperplasia in diabetics differs from non-diabetics [7,11,12]. Diabetic vascular smooth muscle cells exhibit increased rates of proliferation, leading to luminal narrowing [11]. Furthermore, restenotic intimal hyperplasia in diabetics has increased macrophage infiltration [11,12], lipid-rich and collagen-rich sclerotic content [8]. Diabetics have more vaso vasorum neovascularisation, leading to intraplaque haemorrhage [12]. Atherosclerotic laesions in diabetics are more prone to rupture [11]. Furthermore, progression of atherosclerotic plaques is accelerated in diabetics [8]. More extensive neointimal hyperplasia and more rapid plaque formation in diabetics leads to more restenosis and less stable atherosclerotic plaques.

**Table 1:** Key processes in restenosis in diabetics; the processes are specified to the elevated (↑) or decreased (↓) factors contributing to the elevated risk of restenosis in diabeticscompared to non-diabetics.

Influence of 4 Key Factors on Restenosis in Diabetics	
Process	Factor

Proinflammatory state	↑ C-peptide
	↑ Resistin
	↑ CRP
	↑ Il-12

Prothrombotic state	↑ P2Y-receptor
	↑ Endothelial damage by O2-radicals

Accelerated and unstable plaque formation	↑ Vascular smooth muscle cell proliferation
	↑ Macrophage infiltration
	↑ Lipid-rich
	↑ Collagen-rich
	↑ Vaso vasorum neovascularization

Haemodynamics	↓ Vessel lumen diameter
	↑ Multivessel disease
	↑ Viscosity

Several factors can account for the more extensive CAD in patients with diabetes. First, diabetic patients are more likely to have (co-) morbidities, among which are cardiovascular risk factors such as the metabolic syndrome. According to the National Cholesterol Education Program (NCEP) Expert Panel ATP-III-criteria (2001) the metabolic syndrome consist of 3 or more of 5 risk factors: hyperglycaemia, hypertension, decreased levels of HDL, elevated levels of triglycerides and abdominal (visceral) obesity [13]. The metabolic syndrome is associated with a proinflammatory and prothrombotic state [7].

Several molecular mechanisms have been implicated in hyperglycaemic-induced endothelial damage: activation of protein-kinase C isoforms, increased hexosamine pathway flux, increased advanced glycation end product formation, increased polyol pathway flux and activation of the proinflammatory nuclear transcription factor kappa-B [7]. All these mechanisms are associated with overproduction of reactive oxygen species, which lead to endothelial dysfunction, decreased vasodilatation and promotion of atherosclerotic plaque formation [7]. Plaque formation is one of the forces behind restenosis.

Peroxisome proliferator activated receptors (PPARs) play an important role in the atherosclerotic process. Especially PPAR-gamma – a subfamily – is potent in reducing plaque inflammation, in inhibiting expression of adhesion molecules and in inhibiting formation of cytokines (e.g. interleukin 12 [IL-12]) [7]. It is known IL-12 is an important factor in the pathophysiology of atherosclerosis and leads to up-regulation of T1-helper response, which is associated with CAD [14]. Wegner et al (2008) demonstrated that IL-12 levels are elevated in type 2 diabetics based on fasting proinsulin due to insulin resistance [14].

Furthermore, insulin resistance leads to upregulation of the P2Y-receptor-pathway, a platelet membrane protein [15].

In patients with diabetes increased levels of C-peptide, a cleavage product of proinsulin, circulate. C-peptide promotes monocyte and T-lymphocyte recruitment into the vessel wall [16]. Walcher et al (2006) showed that in early arteriosclerotic lesions of diabetic subjects, C-peptide colocalized adherent to vascular smooth muscle cells (VSMCs) in the media [16]. C-peptide induced phosphorylation of Src-kinase, as well as activation of the extracellular signal-regulated kinase 1/2 (ERK 1/2) and phosphatidylinositol 3-kinase (PI3K) leading to increased cell proliferation.

Another pathway involved in proliferation of VSMCs is through the effects induced by resistin, an adipokine, which is elevated in diabetes [17]. One study has shown that resistin has a proinflammatory effect on endothelial cells [17]. Calabro et al (2004) showed that VSMC proliferation was induced by resistin [17]. Resistin induces VSMC proliferation through both ERK 1/2 and Akt signaling pathways. Furthermore, On et al (2007) demonstrated that elevated preprocedural serum resistin levels may prove to be a useful biological marker for coronary artery disease and restenosis in patients with type 2 diabetes [18].

## Coronary Interventions and Restenosis

### Coronary stents

The long-term benefit of balloon angioplasty is mainly limited by restenosis of the treated segment, occurring in one third to half of all treated patients [19]. Restenosis causes recurrent myocardial ischemia and therefore necessitates additional revascularisation [19]. Initially, bare metal stents (BMS) were introduced to overcome flow limiting dissections following balloon angioplasty. In addition it was shown that stents resulted in a lower rate of restenosis and recurrent ischemia. Stents prevent vessel recoil and negative remodeling – important steps in luminal narrowing – and thereby reduce the possibility of restenosis [20]. Intimal hyperplasia, another important step in luminal narrowing, is not prevented by stenting and is the main reason for in-stent restenosis.

Drug-eluting stents (DES) are covered with polymers of immunosuppressive agents e.g. paclitaxel, sirolimus, and zotarolimus, which are progressively eluted the first weeks after implantation. DES treatment in both diabetics and non-diabetics leads to a reduction of target vessel revascularization (TVR) and restenosis after PCI in comparison to BMS [10]. A recent meta-analysis by Stettler et al (2009) demonstrated that in 3852 diabetics and 10947 non-diabetics DES and BMS have comparable hazard ratios for mortality [21]. Furthermore, this study confirmed that DES lead to a decrease in revascularization rates compared with BMS in people both with and without diabetes.

Jensen et al (2009) compared paclitaxel eluting stents and sirolimus eluting stents in 74 diabetics and found that paclitaxel eluting stents had increased lumen reduction [22]. Fröbert et al (2009) compared four types of drug eluting stents in 8,231 patients with diabetes, and found significant differences in restenosis rates, favouring the paclitaxel-eluting stents [23]. However, Lee et al (2009) demonstrated in the Drug-Eluting-Stents in Diabetes (DES-DIABETES) trial with 400 patients that on the long-term the sirolimus-eluting stent lead to a reduction of restenosis, TVR and major adverse cardiac events (MACE) [24]. These results were confirmed by a meta-analysis of 16 randomized trials by Schömig et al (2007) [25].

### Bare metal stent versus coronary artery bypass grafting

In non-diabetics there is no difference in mortality between PCI with BMS and coronary artery bypass grafting (CABG) for multivessel disease, whereas in diabetics CABG is thought to have a favourable outcome in comparison with PCI [26]. The Bypass Angioplasty Revascularization Investigation (BARI)-trial (2007) demonstrated that in 1,476 non-diabetics there was no difference between elective PCI and CABG, for both short-term and long-term survival, with a 10-year mortality rate of 23.0% in the PCI group and 22.7% in the CABG group. However, among the subgroup of patients with treated diabetes (n = 353), CABG had a long-term benefit over PCI, based on reduced cardiac mortality in the patients undergoing CABG, with mortality rates of PCI versus CABG of 54.5% versus 42.1%, respectively [26]. The recent meta-analysis by Hlatky et al (2009) showed that in 10 trials with 7,812 patients confirmed the long-term benefit of CABG [27]. In the 1,233 patients with diabetes the 5-year mortality rate was significantly lower with 12.3% in the CABG group versus 20.0% in the PCI group. In the recent Bypass Angioplasty Revascularization Investigation 2 Diabetes (BARI 2D) (2009) with 2368 type 2 diabetes patients the absolute rates of major adverse cardiac events were equal in patients treated with PCI (n = 798) or CABG (n = 378), i.e. 23% versus 22.4% [28]. In the BARI 2D both BMS and DES were used.

### Drug eluting stents versus coronary artery bypass grafting

Conversely, when comparing DES with CABG for multivessel disease, the Synergy between PCI with Taxus and Cardiac Surgery (SYNTAX) trial by Serruys et al (2009) with 1800 patients showed that the rates of myocardial infarction and mortality were similar between PCI and CABG groups [29]. Major adverse cardiac and cerebrovascular events (MACCE) were significantly increased in the PCI group, mainly due to higher rates of revascularization. In the SYNTAX patients with more severe coronary artery disease, and especially those with diabetes, CABG was related to a higher freedom from re-intervention. These results were corroborated by Tarantini et al (2009), Daemen et al (2008), Briguori et al (2007) and Lee et al (2007), respectively including 220, 159, 218 and 205 diabetics [30-33].

## Antiplatelet Agents

### Thienopyridines

Insulin resistance leads to upregulation of the P2Y-receptor-pathway, a platelet membrane protein [15]. The P2Y-receptor is the target of the active metabolite of clopidogrel. Post-PCI this results in increased platelet reactivity in diabetics [15]. Therefore, a higher dose of thienopyridines seems needed in diabetics to attain an adequate antithrombotic regiment [34]. Illustrating the importance of antithrombotic therapy in diabetics, Stettler et al (2009) demonstrated that, in contrast to non-diabetics, drug eluting stents in diabetics are only safe to use if adequate antiplatelet therapy is administered [21]. Wiviott et al (2008) demonstrated in 3,146 patients with diabetes that prasugrel compared with clopidogrel leads to a reduction in cardiovascular death, MI and stroke (12.2% versus 17%) [35]. This illustrates the potential to overcome the relative antiplatelet drug resistance of diabetes with novel pharmacologic agents.

### Glycoprotein IIb/IIIa receptor blockade

During PCI both profound vessel wall injury and plaque rupture occur. This triggers activation of the coagulation cascade, and adhesion, activation and aggregation of platelets. Platelets express more glycoprotein (GP) IIb/IIIa receptors in diabetics and have a tendency to aggregate, especially in the presence of hyperglycemia [1,36]. Type 2 diabetics on insulin have greater platelet aggregation than type 2 diabetics non treated with insulin [36]. Labinaz et al (2002) compared GP IIb/IIIa receptor blockers in PCIs in diabetics (n = 466) and non-diabetics (n = 1,595) [37]. The group of patients receiving GP IIb/IIIa receptor blockers had significant decreased rates of mortality, TVR and MI. However, the relative risk reduction for diabetics was similar to the reduction in non-diabetics.

## Glucose Lowering Agents and Restenosis

The effect of treatment with glucose lowering agents on macrovascular disease is not well established. In the European Heart Survey a pronounced decrease in cardiovascular events in patients with newly detected diabetes prescribed glucose lowering agents compared with those not receiving such treatment was observed [38]. Several retrospective analyses of databases showed differences in coronary revascularization and MI in patients treated with different regimens (table 2). The major recognized limitation of these analyses is the bias of patients with a higher risk profile treated with insulin or combination therapy [39]. The above-mentioned BARI 2D also showed that after a follow-up period of more than 3 years in patients treated with PCI that no survival benefit could be observed between patients treated with metformin or TZDs (insulin sensitization, 10.2%) as compared to insulin or sulfonylurea (insulin provision, 11.4%) [28]. However, the rate of major cardiovascular events was 3.8% lower in the insulin sensitization group treated with PCI, albeit non-significant. The effect of the individual glucose lowering agents was not reported (yet). The effect of glucose lowering, i.e. more strict versus moderate glucose control, on outcome has been extensively investigated. In a meta-analysis (2009) of 5 large trials the rate of non-fatal myocardial infarction was significantly lower in the stricter compared to the moderate glucose control group, 10.0% versus 12.3% [40]. Whether strict glucose control, irrespective of specific glucose lowering agents, is related to a reduction of restenosis is not known.

**Table 2:** Elevated (↑) or decreased (↓) risk of restenosis, target laesion vascularization (TLR), death or myocardial infarction (MI) of the different groups of glucose lowering agents after PCI.

Risk Modulation of Glucose Lowering Agents in Diabetics After PCI				
Agent	Restenosis	TLR	Death	MI

TZDs	↓	↓	↓	-

Sulfonylurea	↑	-	↑	-

Biguanides	-	↓	-	↓

Insulin	↑	↑	↑	↑

### Thiazolidinediones

Thiazolidinediones (TZDs) are peroxisome proliferator activated receptors (PPAR)-gamma agonists. They decrease insulin resistance, levels of CRP, fibrinogen, LDL-cholesterol, triglycerides and resistin in comparison with placebo [17,41]. TZDs inhibit VSMC proliferation, ameliorating intimal hyperplasia and restenosis [42]. Under high glucose conditions the inhibitory activity of VSMC proliferation by pioglitazone and rosiglitazone is enhanced, in contract to troglitazone [42].

Several studies have suggested the preventive effects of TZDs on atherosclerosis, restenosis, in-stent restenosis, and reocclusion after PCI in patients with type 2 diabetes. Takagi et al (2002) showed in a randomized study that in 55 patients with 60 lesions troglitazone was associated with reduced angiographic in-stent restenosis and neointimal tissue proliferation [43]. Choi et al (2004) found in a case-control study with 83 patients treated with rosiglitazone that at 6 months a significant reduction in restenosis rate, greater minimal lumen stent diameter and albeit non-significant lower TVR [44]. Nishio et al (2006) observed in randomized study with 54 patients that treatment with pioglitazone attenuated neointimal thickening regardless of the type of PCI and resulted in less late luminal loss [45]. The meta-analysis of Rosmarakis et al in 2007 already showed that TZDs decrease the need for target vessel revascularization when 5 trials with 235 patients were analyzed [46]. The meta-analysis by Geng et al published in 2009 consisted of 8 trials with 366 patients and showed that TZDs were associated with a significant reduction in the risk of in-stent restenosis in both diabetic and non-diabetic patients [47]. They also observed a reduction in late lumen loss, percent diameter stenosis, neointimal area/volume, and target lesion revascularization. Recently, Finn et al (2009) randomly assigned 65 diabetics to rosiglitazone or placebo and could not confirm the above described results on luminal loss [48]. An interesting observation was made by Nishio et al (2006), i.e. that in 38 type 2 diabetics similar restenosis rates were present in patients treated with either a BMS with pioglitazone or sirolimus-eluting stent without pioglitazone [49]. Fang et al (2007) demonstrated that rosiglitazone therapy in comparison to standard antidiabetic therapy without rosiglitazone in type 2 diabetics leads to a significant decrease in restenosis and mortality [50]. However, the intervention group significantly used more insulin and biguanides, which might lead to bias of the observed effects. In a study with 417 patients referred for stress testing to evaluate chest pain by Kapinya et al (2008), 222 were treated with conventional therapy only (insulin and insulin secretagogues) and 195 as being treated with insulin sensitizers (metformin and thiazolidinediones) [51]. The rate of ischemia and MI was not significantly lower in patients treated with thiazolidinediones. In the Pioglitazone Effect on Regression of Intravascular Sonographic Coronary Obstruction Prospective Evaluation (PERISCOPE)-trial, Nissen et al (2008) demonstrated that in patients with type 2 diabetes and coronary artery disease treatment with pioglitazone compared with glimepiride results in a significantly lower rate of progression of coronary atherosclerosis, as measured with intravascular ultrasound [52]. The patients receiving pioglitazone also had significantly reduced levels of CRP, triglycerides and HbA1c and an increase in HDL. Nonetheless, this trial did not show any significant differences in clinical outcome between pioglitazone and glimepiride. Recently, Clementi et al (2009) demonstrated in 25 patients with diabetes that administration of pioglitazone combined with atorvastatin leads to a significant regression of coronary atherosclerosis [53].

### Biguanides

The precise mechanism of action of biguanides, i.e. metformin, is not completely elucidated. Metformin has insulin sensitizing effects, and may therefore have cardiovascular effects [54]. In the previously described study by Kapinya et al (2004), only the 125 patients on metformin had lower rates of ischemia and MI [51]. In diabetics undergoing PCI, Kao et al (2004) showed that metformin-therapy in comparison with insulin and sulfonylurea was associated with a reduced risk of MI and mortality [54]. It is not clear whether the superiority of metformin therapy in this trial is due to beneficial effects of the metformin or is due to possible adverse effects of the sulfonylurea on atherosclerosis. In the study by Walker et al (2008) it was observed that patients treated with metformin were less likely to need revascularization or suffer from MI compared to other treatment regimens [39]. Comparable results were observed by Casscells et al with 232,000 diabetics (2008) [55]. On the other hand in a post-hoc analysis of the Diabetes and Insulin-Glucose Infusion in Acute Myocardial Infarction (DIGAMI)-2 trial with 1181 patients no differences on cardiovascular mortality were observed, although metformin was associated with a significant lower rate of non-fatal MI and stroke as compared to sulfonylurea or insulin treatment [56].

Adversely, metformin therapy may also lead to progression of coronary artery disease. Chronic use of metformin leads to decreased intestinal absorption of group B vitamins and folate. These deficiencies lead to elevated levels of homocysteine, which accelerates progression of cardiovascular disease due to adverse effects on platelets, endothelium and clothing factors [57].

### Sulfonylurea

Sulfonylurea act through binding to the sulfonylurea receptor on the surface of pancreatic β cells. This results in closure of the potassium channels and thereby depolarization of the cell membrane and, in turn, opening of voltage-dependent calcium channels. The influx of calcium causes microtubules to contract and the exocytosis of insulin from vesicles. Garratt et al (1999) report that sulfonylurea drugs are associated with an increased risk of in-hospital mortality among 185 diabetic patients undergoing angioplasty for acute myocardial infarction [58]. No association between sulfonylurea drugs and *late *adverse events after primary PCI has been observed. The explanation for this early risk is not an increase in ventricular arrhythmias, but reflects the deleterious effects of sulfonylurea drugs on myocardial tolerance for ischemia and reperfusion. Blockade of K-ATP-channels, which is the target of sulfonylurea drugs, promotes insulin release in pancreatic B-cells, but also affects in certain sulfonylurea drugs cardiac tissue and coronary arteries [59]. This leads to greater vulnerability of the myocardium to ischemia, which results in the increased clinical manifestations of ischemia [57,60].

Sulfonylurea drug use lead to elevated IL-12 serum levels in type-2 diabetics, induced by peripheral insulin resistance and beta cell dysfunction, as expressed by fasting serum proinsulin levels. Therefore, according to the hypothesis by Wegner et al, sulfonylurea drugs can promote atherosclerosis [14].

### Insulin

Huang et al demonstrated that high levels of insulin are potent in stimulating VSMC proliferation [61]. The clinical relevance of this observation has yet to be investigated. In all the above-described studies patients treated with insulin had higher rates of either ischemia or infarction.

## Conclusion

Diabetes is a major risk factor for adverse events after PCI. Diabetics have higher rates of mortality, myocardial infarction and target vessel revascularisation than non-diabetics. Recent therapeutical antithrombotic and percutaneous developments improved the prognosis both for diabetics and non-diabetics, thereby maintaining a worse prognosis for diabetics.

The exact mechanism of restenosis is not yet completely understood, although several contributing factors have been identified. Several glucose lowering drugs influence the restenosis process. Current data of especially metformin and thiazolidinediones indicate beneficial results as compared with insulin and sulfonylurea. However, no large prospective trials in which the clinical impact of these drugs on restenosis is investigated have been undertaken.

The prognosis of diabetics undergoing PCI remains worse than non-diabetics. Possibly the best way to improve the prognosis of diabetics undergoing PCI is to prevent diabetes or to pursue adequate diabetes regulation.

### Future area of interest

Due to the negative influence of diabetes on prognosis after PCI, glucose lowering therapy might have a substantial influence on coronary restenosis and the worse prognosis of diabetics. Future studies might determine whether or not the type of glucose lowering agent has influence on restenosis after PCI.

## Competing interests

The authors declare that they have no competing interests.

## Authors' contributions

The authors responsibilities were as follows – CL: study design, data interpretation, data collection and drafting the manuscript; BR and IH: study design, data interpretation and critical revision of the manuscript for important intellectual content; JM and FZ: study design and critical revision of the manuscript for important intellectual content. All authors approved the final manuscript.
